# Single-molecule tracking to determine the abundances and stoichiometries of freely-diffusing protein complexes in living cells: Past applications and future prospects

**DOI:** 10.1063/5.0155638

**Published:** 2023-08-17

**Authors:** Joshua Robert Prindle, Olivia Isabella Christiane de Cuba, Andreas Gahlmann

**Affiliations:** 1 Department of Chemistry, University of Virginia, Charlottesville, Virginia 22904, USA; 2 Department of Molecular Physiology and Biological Physics, University of Virginia School of Medicine, Charlottesville, Virginia 22903, USA

## Abstract

Most biological processes in living cells rely on interactions between proteins. Live-cell compatible approaches that can quantify to what extent a given protein participates in homo- and hetero-oligomeric complexes of different size and subunit composition are therefore critical to advance our understanding of how cellular physiology is governed by these molecular interactions. Biomolecular complex formation changes the diffusion coefficient of constituent proteins, and these changes can be measured using fluorescence microscopy-based approaches, such as single-molecule tracking, fluorescence correlation spectroscopy, and fluorescence recovery after photobleaching. In this review, we focus on the use of single-molecule tracking to identify, resolve, and quantify the presence of freely-diffusing proteins and protein complexes in living cells. We compare and contrast different data analysis methods that are currently employed in the field and discuss experimental designs that can aid the interpretation of the obtained results. Comparisons of diffusion rates for different proteins and protein complexes in intracellular aqueous environments reported in the recent literature reveal a clear and systematic deviation from the Stokes–Einstein diffusion theory. While a complete and quantitative theoretical explanation of why such deviations manifest is missing, the available data suggest the possibility of weighing freely-diffusing proteins and protein complexes in living cells by measuring their diffusion coefficients. Mapping individual diffusive states to protein complexes of defined molecular weight, subunit stoichiometry, and structure promises to provide key new insights into how protein–protein interactions regulate protein conformational, translational, and rotational dynamics, and ultimately protein function.

## I. INTRODUCTION

Inside the cytoplasm of living cells is a highly concentrated and complex mixture of biomolecules that dynamically interact with one another in space and time. The chemical and biophysical properties of any biomolecule dictate its interactions with other biomolecules and, ultimately, its function. Proteins facilitate most biological processes in a cell, and, while some proteins may act independently, the vast majority of proteins interact with others to enable their function.^1^ A well-known example is the ribosome, which, in its fully assembled functional state, is a complex containing more than 50 individual proteins that binds to mRNA to synthesize new proteins.^2^ In addition to these very large macromolecular complexes, which are stable under most conditions, the cell contains a myriad of smaller proteins and protein complexes with molecular weights less than 200 kDa (i.e., less ∼5 nm in diameter).^3^ In fact, the majority of cellular processes rely on transient protein interactions.^3,4^ Experimental approaches that can quantify to what extent a given protein participates in homo- and hetero-oligomeric complexes of different size and subunit composition are therefore critical for understanding how cellular physiology emerges from the underlying molecular interactions.

There are a number of methods to detect protein–protein interactions between (known) binding partners, and the method of choice is dependent on the stability of the interaction. To identify protein binding partners, co-immunoprecipitation (co-IP),^5^ pull-down assays,^6^ and crosslinking coupled with mass spectrometry^7,8^ can be employed. Complementing these approaches are methods, such as fluorescence/bioluminescence resonance energy transfer (FRET/BRET)^9,10^ and proximity ligation assays (PLA),^11^ that provide ensemble-averaged readouts that depend on the relative distance between two (fluorescent) probes. In addition to these distance-dependent readouts, methods that determine the mode of protein motion, such as single-molecule tracking, fluorescence correlation spectroscopy (FCS), and fluorescence recovery after photobleaching (FRAP), are also useful for detecting interactions with other proteins or other biomolecules within living cells. For example, proteins and protein complexes can bind to stationary cellular targets (such as bacterial flagellar motors^12,13^ and centrioles^14^), translocate in a directional manner along polymerized biomolecules (such as cytoskeletal filaments,^15,16^ DNA,^17,18^ or oriented strands of cell walls^19,20^), or diffuse randomly within the cytosol,^21,22^ within the cell membrane,^23^ or within subcellular domains.^24–26^ Of course, combinations of these different modes of motion are also possible, a key example being transcription factors searching for their cognate target sequences on DNA.^27^ In addition to engaging in interactions with cognate binding partners, proteins also encounter functionally unrelated cellular components through non-specific, transient interactions, such as simple collisions or charge-mediated interactions, as they explore the cellular cytoplasm.^28^ The sum total of these molecular interactions determine the modes of motion of proteins and protein complexes.^3^

The motion types in the above examples can be broadly placed into two categories: Brownian or normal diffusion and anomalous diffusion. The 3D motion trajectories of proteins and small protein complexes that diffuse freely in intracellular aqueous environments are described well by Brownian motion models.^29–32^ For the purpose of this review, free diffusion refers to proteins and protein complexes that do *not* specifically bind to stationary or directionally moving biomolecules and only engage in nonspecific interactions/collisions with other cellular components as they explore intracellular aqueous environments. Anomalous diffusion is observed if the biomolecules of interest interact specifically with cellular structures, such as cytoskeletal filaments/motors or DNA/RNA, or if biomolecules diffuse in actively migrating cells. Anomalous diffusion behavior due to transient confinement and/or directional transport have been extensively reviewed elsewhere.^33,34^ In this review, we instead focus on the Brownian motion of freely-diffusing proteins in intracellular aqueous environments.

The Stokes–Einstein diffusion theory describes how the translational diffusion coefficient *D* of a spherical Brownian particle changes with particle size *r*, temperature *T*, and viscosity *η* of the medium, i.e.,

$$D=k_{B}T/\left(6\pi \eta r\right),$$

where *k*_*B*_ is the Boltzmann constant. Given that the diffusion coefficient scales with the size of the particle, proteins and protein complexes of different size should diffuse at different rates in living cells. Therefore, a freely diffusing protein will be in different diffusive states (each state characterized by a different diffusion coefficient) depending on the proteins or protein complexes it is bound to. Under the assumption that folded proteins and protein complexes can be treated as spherical particles of constant mass density, the particle size can further be related to the molecular weight *MW*, as $r\propto MW^{\frac{1}{3}}$, so that $D\propto T/\left(\eta \cdot MW^{\frac{1}{3}}\right).$ The Stokes–Einstein diffusion theory thus predicts the intuitively expected result that the translational diffusion of proteins and protein complexes decreases as molecular weight increases. This analysis raises an intriguing question: *Is it possible to “weigh” soluble proteins and protein complexes in living cells by measuring their diffusion coefficients?*

The diffusion of proteins in living cells can be measured through either coordinate-targeted or coordinate-stochastic approaches. In coordinate-targeted approaches, such as fluorescence recovery after photobleaching (FRAP) and fluorescence correlation spectroscopy (FCS), fluorescence intensity changes are measured at stationary coordinates and used to infer protein diffusion rates through the observation volume. In FRAP, an area of the biological sample is first photobleached. The fluorescence recovery time, i.e., time needed for unbleached fluorophores to fill the observation volume, is correlated with the diffusion rate of these fluorophores. However, to infer the diffusion coefficient *D* from the recovery time, the properties of the excitation beam and the photophysical properties of the fluorophore must be known and controlled. Failure to account for differences in the laser beam shape across different experiments can lead to high variability in calculating the diffusion coefficient. Additionally, photobleaching of fluorescent molecules during the recovery period can lead to underestimation of the diffusion coefficient.^35^ For these reasons, FRAP data are not typically analyzed to provide quantitative estimates of diffusion coefficients. Instead the time scale of recovery often provides the desired biological insights. For example, if proteins cannot cross the boundaries of liquid–liquid phase separated volumes, the fluorescence recovery time within the bleached phase separated volume will be slow.^36^ FCS relies on the principle that fluorescence intensity fluctuates as single fluorescent molecules diffuse in and out of an observation volume. This fluctuating signal can be correlated with time-shifted replicas of itself to obtain an autocorrelation function (ACF). The shape of the ACF is governed by *any* process that results in fluctuations in the fluorescent signal. Importantly, different processes contribute to the ACF in different time regimes; among these, rotational diffusion of fluorophores primarily contributes at time shifts of 10^−9^ to 10^−7^ s, while translational diffusion contributes primarily at 10^−4^ to 10^−2^ s time shifts. While FCS was originally implemented on a confocal microscope,^37^ many variants have since been developed to study protein diffusion across these timescales. Similar to FRAP, the FCS observation volume is very instrument dependent and needs to be properly controlled. Furthermore, FCS operates in a narrow concentration window to ensure a sufficient number of discernible fluctuations. While modern research microscopes provide single-molecule sensitivity, FCS and FRAP measure fluorescence intensity fluctuations at fixed spatial coordinates over time. Thus, these coordinate-targeted approaches do not track single molecules and, therefore, do not have the ability to provide information on individual protein’s mode of motion. Instead, coordinate-targeted approaches provide an averaged picture of the diffusion of many proteins. For in-depth information on FRAP and FCS, we refer the reader to recent reviews.^38–40^

Coordinate-stochastic localization approaches enable single-molecule tracking by linking successive localizations from the same single molecule into a trajectory. From those trajectories, biomolecular modes of motion in living cells can be determined.^28,41,42^ The power of single-molecule localization and tracking methods derives from the fact that the primary experimental observables are precisely the molecular motions described above, probed at experimentally achievable resolutions. These methods can be broadly placed into two categories: point-spread-function (PSF)-based localization and modulation-enhanced localization. For PSF-based localization methods, such as PALM^43,44^ and STORM,^45^ the diffraction-limited image of a point source (i.e., the PSF) is analyzed to estimate the emitter position.^46–50^ In modulation-enhanced localization methods,^51^ such as MINFLUX,^52–55^ pulsed interleaved MINFLUX (*p*-MINFLUX),^56^ single-molecule confocal laser tracking combined with fluorescence correlation spectroscopy (SMCT–FCS),^57^ orbital scanning,^58–61^ 3D-DyPLoT,^62,63^ 3D-SMART,^64^ and TSUNAMI,^65^ spatially structured illumination patterns result in fluorescence intensity modulations that are analyzed to localize and track individual fluorophores. For freely diffusing molecules, the localization precision of PSF-based and modulation-enhanced localization is comparable, namely, ∼20–60 nm. However, the temporal resolution of modulation-enhanced localization can reach 0.1 ms, which is about two orders of magnitude higher than what is typically achieved in PSF-based localization methods. The increased time resolution of modulation-enhanced localization methods derives from a more efficient use of the information delivered by each photon.^52^ Therefore, modulation-enhanced localization can record single-molecule trajectories containing a larger number of localizations (up to 10^2^–10^3^) compared to PSF-based tracking (up to 10^1^–10^2^). However, tracking of fast diffusing (>5 *µ*m^2^/s) single-molecules in large volumes is experimentally challenging (due photon emission rate limitations of currently available fluorophores) and has not yet been demonstrated with modulation-enhanced localization methods.^66,67^ An advantage of PSF-based localization methods is their high parallelizability: 10^4^–10^5^ trajectories can be obtained in a single experiment. Modulation-enhanced localization methods, on the other hand, typically acquire 10^2^–10^3^ individual trajectories sequentially in time.

Independent of the experimental tracking method, tracking of stationary and/or directionally moving single molecules can provide bound-state lifetimes and molecule velocities, respectively, without the need for extensive data analysis. By contrast, more sophisticated motion models have to be employed to describe the motion of freely-diffusing molecules. In this review, we focus specifically on the use of single-molecule tracking to resolve distinct diffusive states of fluorophore-labeled proteins that manifest in intracellular aqueous environments, as well as on experimental efforts to map these simultaneously occurring diffusive states to specific multiprotein complexes that form in living cells. First, we compare and contrast different data analysis methods that have been developed in recent years to identify, resolve, and quantify different diffusive states, placing a particular emphasis on assumptions made in each analysis method. Next, we discuss additional experimental design approaches that can help the interpretation of the results provided by the analysis of single-molecule tracking data, placing a particular focus on determining the composition of freely diffusing protein complexes. Finally, we discuss the prospect of how single-molecule tracking could be used to not just determine the composition of freely diffusing protein complexes in living cells, but also their molecular weights and stoichiometries.

## II. ESTIMATING PROTEIN DIFFUSION RATES

Determining the rate at which a protein diffuses in living cells is non-trivial. In this section, we first introduce foundational work that revealed how errors, inherent to single-molecule tracking, and systematic factors, such as spatial confinement, can bias the estimation of diffusion coefficients. We then compare and contrast more recently developed data analysis methods that aim to identify, resolve, and quantify the diffusion coefficients of freely-diffusing proteins in intracellular aqueous environments. We place a particular emphasis on the physically relevant parameters that are estimated and the assumptions made in each analysis method. Finally, we review recent implementations of deep learning approaches to characterize the mode of motion of diffusing proteins in a more general manner.

### A. MSD analysis

**FIG. 1.**
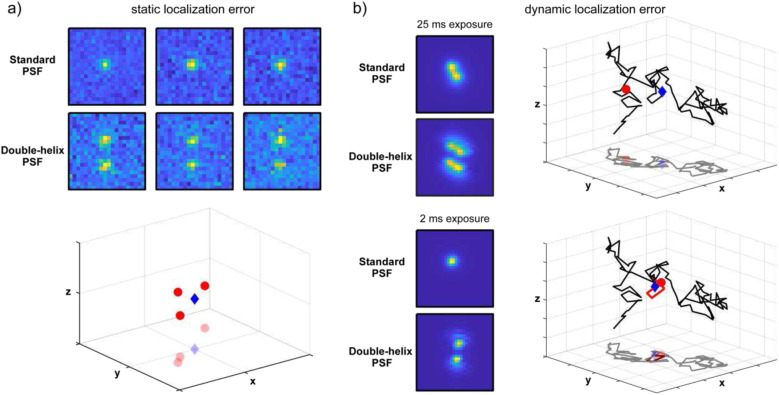
Experimental considerations affecting single-molecule tracking results. (a) For a stationary fluorophore at a fixed position in space (blue diamond), different PSF images will be obtained at different times due to uncorrelated Gaussian camera read noise and Poisson shot noise. (Two sets of simulated images are shown for the standard PSF and the double-helix PSF, respectively.) Because of these random noise contributions, the estimated emitter positions (red circles) will differ from the actual position of the emitter. (b) For a freely diffusing fluorophore, motion-blurred PSF images will be obtained. (Two sets of simulated images are shown for the standard PSF and the double-helix PSF, respectively. Gaussian camera read noise and Poisson shot noise are omitted here for clarity.) Because of motion blur, the estimated emitter position (red circle) will differ from the center-of-mass position of the emitter during the exposure/illumination time (blue diamond). By reducing the exposure/illumination time from 25 to 2 ms (referred to as stroboscopic illumination), the amount of motion blur can be reduced.

Historically, single-molecule trajectories have been analyzed by computing the mean squared displacements (MSDs) at different time lags

$$MSD_{n}=\frac{1}{N-n}\overset{N-n}{\underset{i=1}{\sum }}{\left(\left|{\vec{r}}_{i+n}-{\vec{r}}_{i}\right|\right)}^{2}\;n=1,\cdots \cdot N-1,$$

where *N* is the number of localizations in the trajectory and ${\vec{r}}_{i}$ are single-molecule positions within that trajectory, typically sampled at a constant time interval Δ*t*. For a randomly diffusing Brownian particle, the MSD is proportional to the translational diffusion coefficient *D*, i.e.,

$$MSD_{n}=2\;dDn\Delta t,$$

where *d* is the dimensionality of the acquired single-molecule localizations (*d* = 2 for 2D tracking and *d* = 3 for 3D tracking). In any experiment, there are two main sources of localization errors that affect single-molecule position measurements. Static localization errors limit the precision of single-molecule position measurements, most notably because of the finite number of detected fluorescence photons^46,47^ [Fig. 1(a)]. In addition, localizations of moving emitters suffer from dynamic localization errors, because the fluorescence signal is motion-blurred [Fig. 1(b)]. Dynamic localization errors limit both the precision and accuracy of single-molecule localizations. The combined localization error *σ* modifies the MSD vs time relationship to $MSD_{n}\left(n;\Delta t\right)=2\;dDn\Delta t+2\;d\sigma^{2}$.^68^ The straightforward relationship between the experimental observable, *MSD*_*n*_, and the quantity interest, *D*, has made MSD analysis an immensely popular approach in the biophysical life sciences. However, many (>1000) trajectory points are needed to reliably estimate (within 10% error) the diffusion coefficient of a single-molecule acquired with experimentally realistic localization errors.^69^ Experimentally acquired single-molecule trajectories typically contain only around 10–1000 displacements, which does not meet the above threshold. If the tracked particles undergo unconfined Brownian motion that is governed by a single diffusion coefficient value, >1000 trajectory points could be obtained by pooling a sufficient number of short trajectories. In such a case, MSD analysis would provide an accurate estimation of the diffusion coefficient. However, the assumption of homogeneous, unconfined Brownian motion is unlikely to hold for protein diffusion in living cells. MSD analysis is therefore not suitable to determine diffusion coefficient(s) of single molecules in living cells.

To overcome the drawbacks of MSD analysis, the field has developed a variety of alternative approaches. These approaches are based on curve-fitting or Bayesian inference methods to analyze pooled trajectory data. A common feature of these methods is that researchers have to define, *a priori*, a model *M* that may describe the data. Conceptually, these models can be further subdivided into model components that describe protein diffusion, *M*_*biophysics*_, and model components that describe instrument-related, technical aspects of how the data were acquired, *M*_*experiment*_. A critical challenge for the field is that different research groups often utilize rather different *M*_*biophysics*_ and *M*_*experiment*_ on top of differing data analysis frameworks. In addition to these statistical and probabilistic data analysis approaches, deep learning-based approaches have been applied to extract and rank features describing single-molecule trajectories without reference to specific predefined models *M*. In the following, we will compare and contrast these different analysis methods and highlight their differences and commonalities, as well as their respective scope of application.

### B. Fitting apparent diffusion coefficient distributions using simulated model functions

**FIG. 2.**
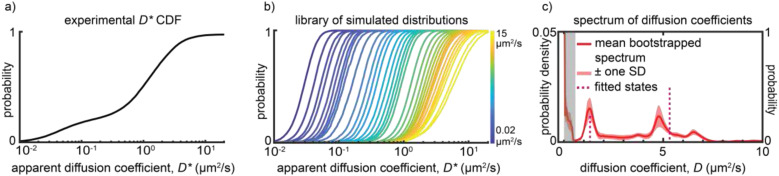
(a) Experimentally measured D* distribution of eYFP-labeled SctQ in *Yersinia enterocolitica*. (b) Simulated distributions of apparent diffusion coefficients based on Monte Carlo simulations of confined Brownian motion in rod-shaped bacterial cells. These simulations account for both random and systematic measurement errors encountered in single-molecule tracking measurements. (c) The diffusion coefficient spectrum (red) of eYFP-SctQ shows two prominent peaks centered at *D* = 1.3 *µ*m^2^/s and *D* = 4.8 *µ*m^2^/s, as well as a smaller peak centered at *D* = 6.5 *µ*m^2^/s. Bootstrapping analysis provides the standard deviation of the spectrum (red, shaded). A two diffusive state model (pink line, dashed) was selected by five-fold cross-validation. The different diffusive states are due to SctQ participating in different hetero-oligomeric protein complexes in the bacterial cytoplasm. SctQ also dynamically binds and unbinds from the membrane-imbedded type III secretion system. Thus, a large stationary population (shaded gray area) is observed as well. Figure adapted from Ref. 32.

The calculated diffusion coefficient from single-molecule *MSD*_*n*_ calculations (i.e., averaged over a single-molecule trajectory) should be regarded as the apparent single-molecule diffusion coefficient D* to distinguish it from the actual diffusion coefficient *D* that governs the motion of the molecule. Estimates of D* differ from *D* because of the drawbacks of MSD analysis, namely, short trajectory lengths and localization errors due to limited photon budgets and motion blurring, but also confinement of trajectories within small volumes, such as bacterial cells,^32,70–74^ cellular organelles,^26^ and phase-separated condensates.^24,25^ Our group developed an approach to account for the complex interdependencies between trajectory length, localization errors, and confinement effects contained in experimental D* distributions [Fig. 2(a)] by simulating D* distributions and then using them to fit the experimental D* distributions.^32,72,74^ For this approach to work, the simulated D* distributions need to be based on a pre-defined model $M=M\left(M_{\mathrm{biophysics}},M_{\mathrm{experiment}}\right)$ that incorporates the above factors as realistically as possible. For bacterial cell imaging, realistically simulated D* distributions can be achieved through a numerical forward convolution approach that begins with Monte Carlo sampling of Brownian motion within a confining volume matching the size and shape of the bacterial cells being imaged. Fine time steps of 100 ns are chosen to generate a list of positions visited by a diffusing molecule during the chosen camera and/or laser exposure time (e.g., 25 ms). The fine trajectory is split up into 50 segments, and the center-of-mass within each segment is computed. The center-of-mass positions are then convolved with the microscope’s PSF to generate 50 subframes, which are summed to generate a motion-blurred image of the diffusing molecule. Next, the signal intensity is scaled to match the experimentally observed signal intensities, and the resulting image is modulated by the addition of background intensity as well as by the addition of Gaussian camera read noise and Poisson shot noise that is inherent to the photon detection process. Simulated localizations and trajectories are obtained through the same localization and tracking algorithms as used for experimentally acquired data, and an apparent diffusion coefficient D* is calculated for each simulated trajectory. This process is repeated for *N* = 5000 trajectories to obtain a well-sampled D* distribution corresponding to a single “true” diffusion coefficient *D*. Through interpolation of a finite set of D* distributions for different diffusion coefficients *D*, a continuous interpolation function is obtained [Fig. 2(b)]. This interpolation function incorporates contributions of dynamic and static localization errors, trajectory length, confinement effects, as well as any biases inherent in the localization and tracking algorithm itself. This interpolation function/model is thus well-powered to fit/explain the acquired experimental data. Diffusive state parameters can be estimated with 20% error for short single-molecule trajectories (less than ten localizations), if *N* = 5000 trajectories are pooled for analysis.^74^

A spectrum of Brownian diffusion coefficients that manifest for tracked single-molecules [Fig. 2(c)] can be generated by fitting a linear combination of apparent diffusion coefficient cumulative distribution functions (D* CDFs) to the experimental CDF.^32^ The fitting procedure begins by generating a D* CDF library at ∼500 different diffusion coefficients *D*_*i*_ in the range of 0.01 to 15 *µ*m^2^/s (based on the above-mentioned interpolation function). A linear combination for experimental data fitting is then constructed according to

$$CDF_{\mathrm{simulated}}=\overset{500}{\underset{i=1}{\sum }}a_{i}CDF_{\mathrm{simulated,i}}\left(D_{i}\right)\;\;\;\;\;\;\;\;\;\;\;\;\;\;\;\;\overset{500}{\underset{i=1}{\sum }}a_{i}=1.$$

A uniform prior probability, i.e., all *a*_*i*_ at an equal initial value, is used as the starting point for fitting *CDF*_*simulated*_ to *CDF*_*experiment*_ using the linear coefficients *a*_*i*_ as adjustable parameters. Plotting the optimal amplitudes *a*_*i*_ as a function of their corresponding diffusion coefficient *D*_*i*_, generates a spectrum of diffusion coefficients for the tracked single-molecules given the time-resolution of the experiment (discussed further below). To assess the uncertainty in each *a*_*i*_, bootstrapping^75^ can be used: the original experimental dataset containing *N* apparent diffusion coefficients is resampled with replacement *n* times and the linear fitting process is repeated for each resampled dataset. For a single molecule whose motion is governed by a single diffusion coefficient *D*, the spectrum will have a distinct peak centered on a specific diffusion coefficient value. For proteins that can become part of larger protein complexes, additional peaks appear in the diffusion coefficient spectrum [Fig. 2(c)].^32^

The peaks and integrated peak areas in the diffusion coefficient spectrum provide the initial values for curve fitting using a more constrained model function

$$CDF_{\mathrm{simulated}}=\overset{K}{\underset{i=1}{\sum }}a_{i}CDF_{\mathrm{simulated,i}}\left(D_{i}\right),$$

$$\overset{K}{\underset{i=1}{\sum }}a_{i}=1,$$

where *K* = 1,2,3, … enumerate the different diffusive states that are resolved in the diffusion coefficient spectrum. Determining the optimal *K*-state model has historically been challenging because optimization routines can get stuck in local minima corresponding to vastly different parameter values that, in some cases, are heavily influenced by the initial parameter values. Spectral peaks that are not clearly resolved can be assigned one or multiple diffusive states as initial guesses to determine the optimal parameters for different *K*-state models. An independent approach to select the optimal *K*-state model that does not overfit the data with too many diffusive states is *m*-fold cross-validation.^76^ In this approach, the data are divided into *m* subsets. One subset is considered the “validation” data while the others are considered “training” data. A model is then fit to the training data and evaluated on the validation data. Generally, as more parameters are added to the model, the cross-validated errors decrease, reach a minimum, and then increase again due to overfitting. When fitting experimental D* CDFs, the optimal *K*-state model can thus be selected based on two criteria: the *m*-fold cross-validation error is comparable to or lower than those of other models, and the model fit parameters agree with the peaks observed in the diffusion coefficient spectrum. This two-step procedure addresses the problem of model selection commonly encountered in frequentist (i.e., least squares- or maximum likelihood-based) curve fitting approaches of single-molecule tracking data.

### C. Analytical diffusion distribution analysis (anaDDA)

**FIG. 3.**
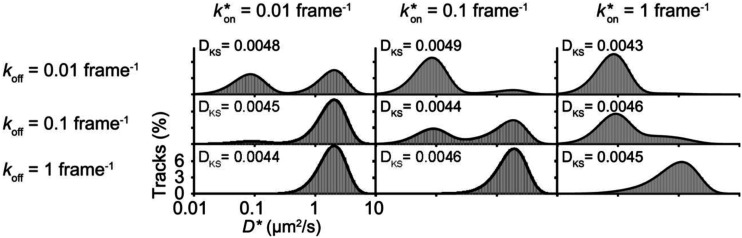
Simulated D* distributions for molecules that switch between a stationary and a freely diffusive (*D* = 4 *µ*m^2^/s) state at the indicated state transition rates. The simulated D* distributions (gray) are fit well to the analytical distributions calculated by anaDDA (black). In the limit of slow transition rates, i.e., k*_ON_ = *k*_off_ = 0.01 transitions per frame, the diffusive states parameters are well-resolved. As the transition rates increase, the D* distributions become skewed. Figure reproduced with permission from Vink *et al.*, Biophys. J. **119**, 1970 (2020). Copyright 2020 Biophysical Society.

In an alternative frequentist approach, Vink *et al.* developed analytical diffusion distribution analysis (anaDDA), which uses an analytically defined model $M\left(M_{\mathrm{biophysics}},M_{\mathrm{experiment}}\right)$ to fit experimental D* distributions.^77^ An important new feature of anaDDA is that spatial confinement of trajectories in spherocylindrical volumes (approximating rod-shaped bacterial cells) is included in the model. Previous analytical approaches were, by contrast, constrained to geometrical volumes, such as spheres^78^ and cubes,^79^ for which analytical solutions were available. The analytical approach of anaDDA allows for facile tuning of model hyperparameters, such as confinement volume shape, frame rate, and illumination times, which may differ across experiments. Facile hyperparameter tuning is a clear advantage over the simulation approach described in Sec. II B in terms of the computational costs required to define the model *M*. anaDDA also has the added capability of inferring transition rates between diffusive states. This additional model component is important for proteins that are likely to bind to and unbind from interaction partners on timescales comparable to or shorter than the durations of the single-molecule trajectories being analyzed. anaDDA thus uses a probability distribution function (PDF) that includes the transition rates, *k*_*ON*_ and *k*_*OFF*_, between diffusive and stationary states (Fig. 3) to fit to experimental D* distributions. This kinetic model component was adapted from probability distribution analysis originally developed to determine the presence of interchanging conformational states within individual single-molecule FRET traces.^80,81^

The capabilities of anaDDA were tested on simulated diffusive particles that transition between stationary and freely diffusing states in a confined volume^22^ corresponding to an *E. coli* cell. Transition rates of 0.01 to 10 transitions per frame were considered at different numbers of displacements per track (1–8) and different frame rates (20–100 Hz). Localization error with a standard deviation of 20–50 nm (drawn from a zero mean normal distribution) was added to each simulated single-molecule position. Dynamic localization errors were not specifically included, as the anaDDA model assumes that the single-molecule data is obtained using stroboscopic illumination, for which motion blur is reduced.^82,83^ With a sufficient number of trajectories (∼10 000), anaDDA was able to accurately extract kinetic parameters, when 0.1–10 diffusive state transitions occurred within a single trajectory. For spheres and rod-shaped cylindrical geometries of different sizes, the confinement corrections in anaDDA further increased the accuracy and precision of the diffusion coefficient estimates of unbound molecules.

The ability to easily analyze data with different frame rates is an additional benefit of anaDDA. Analysis of combined trajectory data with respective frame times of 10 and 50 ms provided more accurate diffusion coefficient and population fraction estimates compared to analysis of trajectory data collected at a single frame time only. Combining and analyzing trajectory data collected at different frame times thus reduces the number of possible parameter values that accurately fit the combined data. This finding is consistent with previous work showing that time-averaged displacement analysis can be used to determine whether the single-molecule trajectories contain information about state switching events that occur on the same time scale as the single-molecule trajectory durations, but not about those that occur on substantially faster or slower time scales.^74^ An important limitation of anaDDA is that it is only able to fit up to a maximum of two diffusive states. If the diffusion coefficients of these states are sufficiently different, then the state transition rates can be accurately determined. On the other hand, if the diffusion coefficients are similar so that their D* distributions overlap extensively (as is often the case for freely diffusing proteins), then the transition rates cannot be determined reliably.

### D. Bayesian inference

**FIG. 4.**
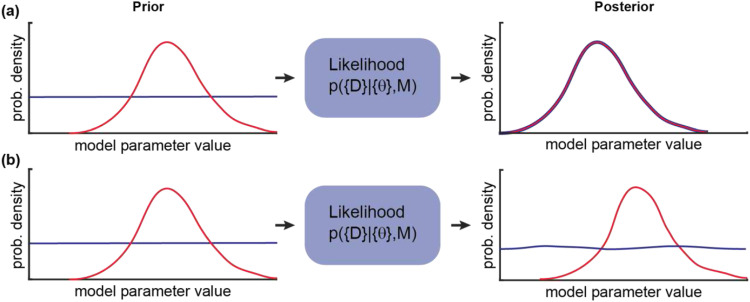
Bayesian inference maps the prior to the posterior through the likelihood. The prior can be a uniform (blue) or non-uniform probability distribution (red), depending on whether any prior knowledge about the parameter values is available. (a) If an experiment is performed that provides substantial new information about the phenomenon being studied, then the likelihood is “strong” and the shape of the posterior will differ from the prior. (b) If an experiment is performed that provides limited new information about the phenomenon being studied, then the likelihood is “weak” and the shape of the posterior will not differ substantially from the chosen prior.

As an alternative to the frequentist curve fitting approaches described above, the field has also begun to employ Bayesian inference methods. Bayesian inference provides an objective framework based on probability theory to estimate diffusive state parameter distributions and select the optimal model, *M*_*biophysics*_. A key distinguishing feature of Bayesian inference is that it provides distributions of diffusive state parameters, namely, the posterior probability density function (the posterior, for short). The posterior quantifies the uncertainties in the model parameters given a model $M=M\left(M_{\mathrm{biophysics}},M_{\mathrm{experiment}}\right)$ that is designed to explain the acquired data $\left\{D\right\}$. According to Bayes’ theorem, the posterior is given by

$$p\left(\left\{\theta \right\}|\left\{D\right\},M\right)=\frac{p\left(\left\{D\right\}|\left\{\theta \right\},M\right)p\left(\left\{\theta \right\}|M\right)}{p\left(\left\{D\right\}|M\right)},$$

where the first term in the numerator, $p\left(\left\{D\right\}|\left\{\theta \right\},M\right)$, is referred to as the likelihood, i.e., probability of observing the data given the parameters $\left\{\theta \right\}$ of a given model *M*. The second term in the numerator, $p\left(\left\{\theta \right\}|M\right)$, is the prior probability density function (the prior, for short) of the parameters $\left\{\theta \right\}$ given the model *M.* At the most fundamental level, Bayesian inference can be seen as the process of mapping the prior to the posterior through the likelihood (Fig. 4). The prior can be a uniform or non-uniform distribution depending on whether any prior knowledge about the parameter values is available, for example, from previous experiments. The term in the denominator, $p\left(\left\{D\right\}|M\right)$, is referred to as the evidence, which quantifies the probability of observing the data $\left\{D\right\}$ given the model *M*. Maximizing the evidence requires selecting the model that best describes the data. In practice, the calculation of the posterior and the evidence is not possible using analytical equations. Thus, numerical methods are employed, most prominently Markov Chain Monte Carlo (MCMC) sampling^84^ and variational methods.^85^ Because numerical methods can be computationally costly, approximations are typically employed to reduce the time required to infer model parameters and perform model selection. For a more complete discussion of Bayesian inference, we instead refer the reader to recent reviews^86^ and textbooks^87^ on this topic.

In addition to earlier work,^88–90^ Persson *et al.* developed variational Bayesian single-particle tracking, vbSPT,^91^ to analyze single-molecule trajectory data. This approach infers the number of diffusive states, as well as their diffusion coefficients, relative population fractions, and the transition rates between those diffusive states. vbSPT performs model selection using a maximum-evidence criterion to determine the number of diffusive states and utilizes a hidden Markov model to determine diffusive state transition rates. When presented with simulated single-molecule trajectories (confined within an *E. coli*-sized spherocylindrical volume), the algorithm correctly inferred the number of diffusive states using a few thousand short trajectories. However, for the correct inference of transition rates, more than 10 000 single-molecule trajectories are required. The diffusive state resolving capability of vbSPT additionally depends on the localization error, how similar the diffusion coefficients are, and how often the states interconvert in relation to the frame rate (consistent with results obtained using frequentist curve fitting approaches^74,77^). Specifically, vbSPT was able to resolve diffusive states having a 50% difference in their diffusion constants, when the average diffusive state lifetimes were ∼10 time steps and >10 000 pooled trajectories were analyzed. When the standard deviation of Gaussian localization errors was increased in simulated trajectories to 30 and 40 nm, the accuracy of the estimated diffusive state relative population fractions worsened, and the transition rates were more heavily biased by the prior—an effect that was attributed to data with increased localization error having an overall lower information content. Thus, the information content in the data is a key limiting factor for Bayesian inference and equally so for frequentist curve fitting approaches. To extract diffusive state parameters accurately using either approach, as many single-molecule trajectories as possible should be acquired with the highest possible spatial and temporal resolution. In practice, it remains difficult to estimate transition rates accurately based on short and noisy single-molecule trajectories.^67^ It is therefore critical that researchers verify how robust the posterior probabilities densities are to variations in the prior. An important drawback of vbSPT is that it does not consider the diffusion coefficient-dependence of the localization error or spatial confinement of single-molecule trajectories. Localization errors for simulated trajectories were drawn from a zero-mean normal distribution of constant width for all trajectories—a simplifying approximation that neglects motion blurring effects for fast moving molecules.

Subsequent Bayesian inference approaches were developed to incorporate localization errors more accurately.^92,93^ Lindén *et al.* used Bayesian inference to determine the localization as well as the localization error from motion blurred and out-of-focus point-spread functions produced by diffusing molecules. The authors incorporated a previous analytical model by Berglund *et al.*^94^ for diffusing molecules into a model including multiple diffusion states governed by a hidden Markov process. Estimation of diffusion coefficients and state switching events was performed not by a full Bayesian inference treatment, but by a variational expectation maximization (EM) algorithm, which provides the most likely parameter values [i.e., the maximum *a posteriori* probability (MAP)] instead of full posterior probability density function. Inclusion of point-wise error estimates based on the experimentally acquired PSFs was shown to improve the accuracy of diffusion coefficient determination and, importantly, more accurate detection of state transitions between simulated *D* = 0.1 *µ*ms^2^/s and *D* = 1 *µ*ms^2^/s diffusive states.

The Berglund *et al.*^94^ model and its explicit treatment of localization errors is also incorporated into a more recent Bayesian inference routine named single-molecule analysis by unsupervised Gibbs sampling (SMAUG).^93^ Gibbs sampling is a particularly efficient approach within the Markov Chain Monte Carlo (MCMC) family of sampling approaches; it samples from conditional probability distributions to estimate a multivariate posterior distribution. Gibbs sampling is useful when it is difficult (or impossible) to sample from the joint probability distribution directly. When tested against a simulated dataset containing a mixture of diffusive states whose diffusion coefficients differed by 60% or more, SMAUG correctly identified the number of diffusive states and provided distributions of diffusion coefficients, population fractions, diffusive state transition rates, as well as localization error distributions for each diffusive state. As was the case for vbSPT, estimation of the transition rates seemed to be most challenging. Also, like vbSPT, SMAUG’s underlying model does not incorporate spatial trajectory confinement. Thus, these algorithms are, in their current forms, applicable to very slow-moving single molecules that can be assumed not to be influenced by confinement effects.

Most recently, Heckert *et al.* used a variational Bayesian framework to analyze single-molecule tracking data that do not originate from a small number of discrete diffusive states.^95^ Instead of trying to infer the correct number of diffusive states, as is done by vbSPT and SMAUG, “only” the population fractions of a large number of diffusive states whose diffusion coefficients are fixed *a priori* are estimated. Displaying the population fractions produced diffusion coefficient spectra similar to those obtained by the linear least squares curve fitting approach used by Prindle *et al.*^32^ An additional component incorporated into the underlying model *M*_*experiment*_ was a specific experimental effect, termed defocalization, which results in under-sampling of fast diffusive states in 2D single-molecule tracking setups. Defocalization occurs because the effective single-molecule detection volume of conventional 2D imaging systems is less than 1 *µ*m thick. Fast-diffusing molecules transit in and out of this detection volume more rapidly than slow-diffusing molecules, resulting in shorter trajectories. The defocalization effect is pronounced when tracking single molecules in large eukaryotic cells and even cell nuclei, but is not a factor in bacterial cells, where single-molecules never go beyond the depth-of-field of the imaging system.^92^ The authors also found that the defocalization effect can be calibrated to adjust the inferred posterior population fraction estimates in a final postprocessing step and therefore does not need to be incorporated explicitly into *M*_*experiment*_ used for Bayesian inference.

In summary, Bayesian inference provides an objective, streamlined framework for model selection and for model parameter and parameter uncertainty estimation. Frequentist curve fitting approaches can achieve the same, but only when combined with statistical treatments, such as bootstrapping and model cross-validation. Bayesian inference methods have expanded on the number and types of diffusive state parameters that can be extracted from pooled single-molecule tracking data, namely, transition rates between diffusive states and localization errors. However, based on the results available to date, it appears that the transition rates between diffusive states remain the most challenging model parameters to infer. Another important limitation of all the aforementioned Bayesian approaches is that their underlying *M*_*biophysics*_ do not consider spatial confinement of single-molecule trajectories. Future work should therefore focus on enabling the analysis of spatially confined molecules that are diffusing fast in intracellular aqueous environments.

### E. Machine learning

In contrast to the frequentist and Bayesian approaches described above, in which the processing procedure is explicitly defined, machine learning algorithms can learn the desired conversion from input (i.e., the single-molecule trajectory data) to predefined outputs from training data. Machine learning algorithms have been applied to classify the type of motion in a single-molecule trajectory, e.g., Brownian diffusion, confined diffusion, or directed motion, among many others.^96–104^ In the anomalous diffusion challenge, which included 13 different machine learning-based approaches and two classical statistics-based methods, the machine-learning approaches were found to be superior in classifying the mode of motion. Despite this success, classification of the mode of motion provides insights into a limited subset of biological problems and is not of immediate use to resolve interactions between freely diffusing proteins in intracellular aqueous environments. Identifying modes of motion requires a clear understanding of why and how a specific motion type may be manifesting in the data, i.e., do they show up because of biological reasons (which should be captured in *M*_*biophysics*_) or due to experimental design (which should be captured in *M*_*experiment*_). To go beyond mere classification of the mode of motion, machine learning algorithms have also been applied to estimate additional parameters, such as the diffusion coefficient and the Hurst exponent for fractional Brownian motion.^97^ All these methods rely on identifying the specific modes of motion with which the network is trained and thus cannot be generally applied.

**FIG. 5.**
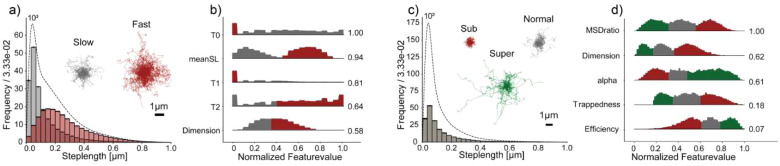
(a) Displacement distribution of freely diffusing, state-switching molecules in slow (gray) and fast (red) diffusive states. Also shown are overlays of 100 randomly chosen trajectories for each diffusive state. (b) Distributions of the five highest-ranking fingerprinting features. (c) Displacement distribution of diffusing molecules governed by different modes of motion: subdiffusion (red), normal diffusion (gray), and superdiffusion (red). Also shown are overlays of 100 randomly chosen trajectories for each mode of motion. (d) Distributions of the five highest-ranking fingerprinting features. Figure reproduced with permission from Pinholt *et al.*, Proc. Natl. Acad. Sci. U. S. A. **118**, e2104624118 (2021). Copyright 2021 National Academy of Science.

To make trajectory analyses more general, single-particle diffusional fingerprinting was developed by Pinholt *et al.*, which uses features obtained from experimentally acquired trajectories as the input for two sequential layers of machine learning to classify and subsequently rank which features are most descriptive of the trajectory, this output being the unique diffusional fingerprint.^105^ Unlike the classifiers used in the past, fingerprinting trains and predicts on experimental data, eliminating the need for simulated datasets that may not explain experimental data properly in all cases. Diffusional fingerprinting centers around the idea that a population of identical diffusers will have a characteristic distribution of the fingerprinting statistics. Classification and ranking is performed as follows: First, a logistic regression classifier considers which of the 17 single-molecule fingerprinting features contributed to the trajectory. These fingerprinting features include statistics from hidden Markov modeling, such as diffusive state and average residence time (τ), as well as MSD analysis, such as mean MSD, diffusion coefficient (*D*), and the MSD power law scaling coefficient (α). In addition, the directionality and symmetry of the trajectory is quantified based on kurtosis, gaussianity, fractal dimension, and track linearity. Second, representation learning automatically ranks the features of relevance for describing the set of trajectories (Fig. 5). The benefits of this approach are that the use of 17 features creates a nuanced description for a dataset, and one can easily decide whether diffusion processes are different between two separate datasets. This approach can generally be applied to a wide range of diffusional phenomena without an *a priori* model or decisions of relevant features by the user. In fact, the authors demonstrate diffusional fingerprinting on a diverse set of systems, including proteins diffusing in live cells and differently coated nanoparticles diffusing in mucus on a lipid membrane.

Although this approach can be applied across multiple biological systems and diffusive phenomena, to interpret the biological context of these differences, it helps if the datasets considered are clearly related, e.g., truncations, mutations, or the presence or absence of signal sequences that determine protein localization and/or function. Overall, diffusional fingerprinting helps uncover subtle but distinctive features of a dataset to achieve a comprehensive description of diffusion that is particularly well-suited for the comparison of related proteins. It is important to note that diffusional fingerprinting was demonstrated on data with 20–1280 displacements per trajectory. It remains to be seen whether this approach is also suited for short single-molecule trajectories with less than ten displacements.

## III. INTERPRETING DIFFUSIVE STATES

As discussed above, there are a range of options available to analyze single-molecule trajectory data and resolve one or more diffusive states. *How, then, should these diffusive states be interpreted?* As explained in Sec. I, the Stokes–Einstein diffusion theory predicts the intuitively expected result that the diffusion of proteins and protein complexes decreases as their molecular weights and sizes increase. Thus, it is reasonable to hypothesize that diffusive states slower than those of monomeric proteins materialize, because these proteins become part of larger homo- or hetero-oligomeric complexes.

It is important to note that single-molecule tracking datasets report “only” on the time-dependent localizations of the labeled proteins of interest, whereas the biological context surrounding the tracked proteins (including specific interaction partners) is not directly probed. While some proteins are very dilute, allowing for dual-color single-molecule tracking,^103,106,107^ most situations require sparse labeling to be able to resolve single molecules. Under these conditions, dual-color tracking experiments to observe two interacting proteins on the same trajectory (which would indicate co-diffusion as a bound complex) are not practically feasible; the probability of observing two proteins in the same space at the same time is negligible given the stochastic nature of single-molecule blinking or photoactivation/photoswitching. Thus, single-color tracking experiments are typically performed, and so the biological significance of the obtained trajectories and resulting diffusion coefficients is not immediately apparent. It is therefore necessary to acquire additional datasets, in which the interacting partners themselves are labeled and tracked, or datasets in which the tracked protein complexes and the individual components thereof are chemically or genetically perturbed. Analyzing the pooled results from many such experiments provides additional biological context and thus allows for better interpretation of any observed diffusive states. In the following, we will describe commonly used experimental perturbations and highlight additional experimental considerations necessary to enable quantitative cross-experiment comparisons of diffusive states observed in different systems or under different environmental conditions.

Performing diffusive state analyses on multiple datasets, in which different potential interacting partners are labeled and tracked independently, can reveal shared diffusive states. Shared diffusive states can be indicative of complex formation, provided that appropriate measures have been taken to control environmental factors that may affect diffusion, such as the temperature (*T*)^108^ and the biomolecular density^109,110^ and composition^111^ of the cytoplasm. The cytoplasmic density and composition are typically controlled by employing the same cell culturing protocols and imaging conditions for different experiments. Once shared diffusive states have been identified across multiple datasets, they can then be evaluated in the context of newly introduced genetic perturbations, such as gene deletions, gene truncations, or point mutations, which disrupt complex formation and thus result in changes in the diffusion coefficient (i.e., new diffusive states). This approach can help identify the exact domains and/or residues that govern protein–protein interactions.^112–119^ When genetic approaches are not feasible, e.g., when the protein of interest performs essential cellular functions, experimentally controllable, inducible and transient perturbations are a better alternative. Optically inducible dimerization systems,^120^ such as Cry2,^121^ iLID,^122^ and MAGNET,^123^ as well as chemically inducible dimerization systems,^124^ such as the rapamycin-dependent FKBP–FRB heterodimerization,^124–126^ enable manipulation of protein spatial localization to specific non-native cellular compartments in so-called knocksideways assays.^127,128^ When combined with single-molecule tracking, manipulating the spatial distributions of otherwise freely diffusing proteins could aid the assignment of protein composition to specific diffusive states.^129^ Specifically, the diffusive state corresponding to a hetero-oligomer would be depleted when either interacting partner is sequestered to a non-native compartment or otherwise dimerized. In addition to manipulating spatial distributions, Graham *et al.* recently described proximity-assisted photoactivation (PAPA), a property of rhodamine dyes, in which one fluorophore reactivates another from a dark state in a distance dependent matter.^130^ When combined with single-molecule tracking, PAPA can be used to enrich trajectories corresponding to double-labeled complexes, which enables identification of interacting or noninteracting molecular subpopulations.

In addition to genetic and opto-/chemo-genetic dimerization-based perturbations, some proteins and protein complexes may be targeted specifically. However, it is important to determine whether targeted perturbations of proteins and protein complexes also result in changes to cellular physiology that generally affect diffusion in intracellular aqueous environments. For example, Oncocin112 is an antimicrobial peptide that binds in the peptide exit tunnel of 70S ribosomes and prevents the transition from initiation to elongation in translation. Thus, upon Oncocin112 treatment, 70S ribosomes get stuck at the start codons resulting in fewer mRNA bound ribosomes compared to untreated cells. Indeed, the diffusion coefficients of 30S ribosomal subunits increased upon Oncocin112 treatment, consistent with fewer 70S polysomes.^131^ However, Oncocin112 treatment also resulted in impeded cell growth and thereby decreased average cell size. This introduces additional factors that need to be considered before single-molecule tracking data from different experiments can be compared. The authors considered the effect of increased cellular confinement in smaller cells, but additionally, the cytoplasmic density may also differ between untreated and Oncocin112-treated cells. Consistent with that possibility, a *D* = 5 → 4 *µ*m^2^/s change in the diffusion coefficient of the free fluorescent protein Kaede was observed. Thus, while single-molecule tracking is a technique that can be applied to a variety of biological systems under different environmental conditions, it is important to consider whether any targeted perturbations also have direct effects on the cellular environment and thereby diffusion.

## IV. DEVIATIONS FROM STOKES–EINSTEIN DIFFUSION THEORY

As diffusion coefficient measurements of proteins become more common, it has become possible to test whether the $D\propto T/\left(\eta \cdot MW^{\frac{1}{3}}\right)$ scaling relation predicted by Stokes–Einstein diffusion theory applies to diffusion in intracellular aqueous environments. Deviations from Stokes–Einstein scaling should be expected because the cytoplasm is not a homogeneous medium of uniform viscosity, but instead is a complex mixture of crowding agents, many of which (e.g., DNA, mRNA, or ribosomes) are substantially larger than the diffusing proteins and protein complexes of interest. Interestingly, currently available results show that, as molecular weight increases, the diffusion rates of proteins and protein complexes is slower than what is predicted by the Stokes–Einstein diffusion theory. One approach to explain this observation is to make the viscosity term dependent on the size of the diffusing molecule.^132,133^ Another approach is to adjust the *D* ∝ *MW*^*β*^ scaling exponent *β*. The results from early, independent experiments clearly show that *β* < −1/3. (We note that this scaling only considers negatively charged proteins. Positively charged proteins exhibit even slower diffusion on average due to their strong electrostatic interactions with negatively charged, quasi-stationary biomolecules.^134^) In a 2010 study,^110^ a scaling exponent *β* of −0.7 was determined using pulsed-FRAP measurements on fluorescently labeled glucose (NBD-glucose, 423 Da), green fluorescent protein (GFP, 26.9 kDa), and a large oligomeric protein assumed to consist primarily of four subunits of GFP-tagged β-galactosidase [(β-gal-GFP)_4_, ∼582 kDa]. When cells were osmotically upshifted (i.e., grown in media supplemented with 2M NaCl), NBD-glucose remained fairly mobile, whereas the diffusion of GFP and (β-gal-GFP)_4_ became comparatively more impeded. In these osmotically upshifted cells, a scaling exponent *β* of −0.8 was determined—an effect attributed to increased biomolecular density in the cytoplasm. In a 2011 review,^135^ FRAP- and FCS-based diffusion coefficients measurements from multiple studies for different-sized proteins were combined and a scaling exponent *β* of −0.7 was determined.

**FIG. 6.**
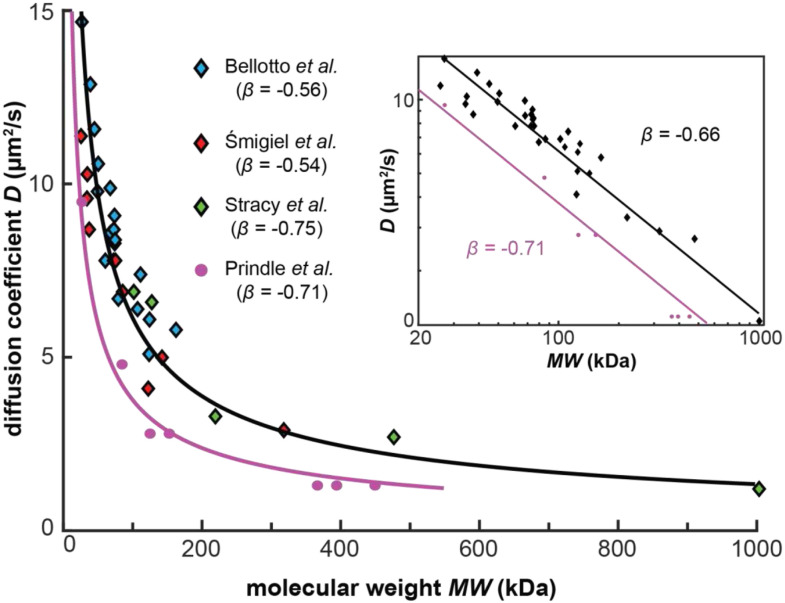
Power-law scaling of diffusion coefficients vs molecular weight data of different proteins and protein complexes. Measurements obtained from three independent studies in *E. coli* (black diamonds)^108,136,137^ results in a scaling exponent *β* of −0.66 (black line). In each study, cells were grown in minimal growth media on the day of imaging. In *Y. enterocolitica* (pink circles), a scaling exponent *β* of −0.71 (pink line) was obtained for freely diffusing proteins in osmotically upshifted cells. Inset: log–log plot of same data.

More recent experiments agree with these findings (Fig. 6). The FCS-measured diffusion rates for sfGFP fusion proteins with molecular weights ranging from 25 to 165 kDa produced a scaling exponent *β* of −0.56.^108^ The tested proteins are globular in shape and are not known to bind DNA or form homomultimers. Also in *E. coli*, single-molecule displacement mapping^111^ was used to study diffusion of fluorescent fusion proteins with molecular weights ranging from 25 to 300 kDa. The proteins were chosen based on the criteria that they do not interact with DNA or with any other proteins. The results showed a scaling exponent *β* of −0.54.^136^ DNA-binding proteins with molecular weights ranging from ∼70 to ∼500 kDa were studied using single-molecule tracking in *E. coli*, in which DNA was enzymatically degraded. These experiments produced a scaling exponent *β* of −0.75.^137^ In contrast to the non-interacting proteins used in the above studies, 3D single-molecule tracking was used to measure the diffusion coefficients of three soluble proteins of the type 3 secretion system (T3SS) in osmotically upshifted *Y. enterocolitica*. T3SS proteins are known to interact with each other to form hetero-oligomeric complexes. When calculating the molecular weights based on the likely complex stoichiometries, a scaling exponent *β* of −0.71 was obtained.^32^

While deviations from Stokes–Einstein diffusion theory can be expected, the observed magnitude, directionality, and reproducibility of this deviation is intriguing. In all the above-mentioned studies, the experimentally measured scaling exponents is substantially larger than the *β* = −1/3 exponent in the Stokes–Einstein diffusion theory. Perhaps even more intriguing is that scaling exponents ranging from −0.54 to −0.8 seem to materialize for different proteins acquired in different (bacterial) species, under different environmental conditions, using different experimental approaches taken by different researchers. These results suggest a conserved property of the cytoplasm and implicate the molecular weight as a major determinant of protein diffusion rate, at least for proteins <200 kDa. With a robustly calibrated *D* vs *MW* scaling relationship in hand, it could thus be possible to “weigh” soluble proteins and protein complexes in living cells by measuring their diffusion coefficients.

Further experiments will help substantiate the observed deviation from the Stokes–Einstein diffusion theory and, perhaps, determine more precise values for the scaling exponent *β*. For example, although the above studies account for effects of confinement on diffusion, the studied proteins were assumed to adopt a single diffusive state (i.e., it was assumed that diffusion was solely dictated by the effects of macromolecular crowding and nonspecific interactions). While the diffusion coefficients were measured for proteins with no known interacting partners (according to *in vitro* biochemical data), this assumption may not hold in the context of living cells. It would therefore be beneficial for future tracking studies to quantify and resolve the diffusive states that may manifest for an individual protein, as discussed in Sec. II. Furthermore, it would be insightful to perform a quantitative comparison of diffusion coefficients obtained at different time scales by FCS and single-molecule tracking for the same proteins within the same cells that are grown and imaged under the same environmental conditions. Such experimental designs would eliminate many biological variables that are known to affect diffusion. Also, to determine the range of molecular weights that can be resolved with diffusion coefficient measurements, additional datapoints for proteins and protein complexes with molecular weights larger than 200 kDa are needed. Such measurements will determine the range over which the same *D* ∝ *MW*^*β*^ scaling can be applied.

The size and shape of proteins and protein complexes could also be systematically varied to determine to what extent these factors affect protein diffusion in intracellular aqueous environments. In this context, the effects of the fluorescent label on protein interactions and protein diffusion could also be considered. Single-molecule localization and tracking experiments in living cells typically utilize fluorescent fusion protein or HaloTag or SNAP-tag fusion proteins that bind functionalized organic dyes. Such protein tags can be of similar size as the tagged proteins of interest, which can result in steric effects that interfere with native protein interactions, as well as slow down diffusion. While it is possible to account for the added molecular weight of a protein label, labeling proteins site-specifically with small organic dyes can be advantageous to avoid both steric perturbations and changes in the labeled protein’s size and shape upon labeling. Site-specific labeling can be achieved using unnatural amino acid incorporation.^138,139^ For example, *trans*-cyclooctene (TCO) or Bicyclo[6.1.0]nonyne (BCN) containing unnatural amino acids rapidly and specifically react with tetrazine-functionalized fluorescent dyes in live cells, resulting in covalent attachment.^139,140^ However, the possibility of off-target labeling, either though misincorporation at undesired amber nonsense codons or nonspecific dye interactions, makes unnatural amino acid labeling a more challenging approach compared to fluorescent or self-labeling fusion proteins.

Testing whether the same *D* ∝ *MW*^*β*^ scaling manifests at different steady-state temperatures and osmotic conditions might reveal additional factors that influence protein diffusion. For example, it was shown that increased metabolic activity fluidizes the bacterial cytoplasm and that the cytoplasm also displays viscoelastic and glass forming properties that influence the motion of very large (>30 nm in diameter) biomolecular complexes.^141^ It remains to be determined to what extent a cell’s overall metabolic activity affects the diffusion of smaller proteins and protein complexes (i.e., those with molecular weights of less than 200 kDa). It also remains to be determined how protein diffusion is impacted by sudden environmental changes. As dynamic living systems, cells likely compensate any short-term changes in cytoplasmic density/fluidity that are introduced by sudden changes in temperature and/or osmotic conditions. It will therefore be beneficial to test how diffusion coefficients change in real time after an experimental perturbation is made. Such measurements will provide biological insights into how cells regulate cytoplasmic fluidity in response to sudden environmental changes.

## V. COMPUTATIONAL SIMULATIONS OF PROTEIN DYNAMICS IN CROWDED ENVIRONMENTS

As the above discussion illustrates, many questions remain about how biological macromolecules behave inside cells where electrolytes, osmolytes, and small molecules, as well as specific and nonspecific biomolecular interactions affect protein conformational, translational and rotational dynamics, and ultimately protein function. The experimental data acquisition and data analysis approaches described in this review can provide single-molecule-specific information about the motion state(s) and, in some cases, the transitions between those motion states of fluorescently labeled proteins on time scales ranging from 100 *µ*s to 1 s. While experimentally measured single-molecule trajectories are undoubtedly affected by the sum total of molecular interactions in the cytoplasm, the biological context that produces these interactions is not accessible by experiment at this time. Computational simulations therefore provide an indispensable complement for linking dynamic biomolecular structures to (cell) biological function.^142^ All-atom molecular dynamics (MD) simulations using highly crowded complex biomolecular mixtures of molecules would reproduce conformational, rotational, and translational dynamics most faithfully, because MD models are fundamentally based on known physical laws governing biomolecular interactions (e.g., hydrogen bonding, van der Waals forces, electrostatic interactions, and hydrodynamic interactions). Given the capability to computationally predict protein structures with high accuracy^143,144^ and the wealth of experimental data available for the molecular composition and structural organization of the cytoplasm, the starting points of MD simulations can be defined with high biological realism. However, whole-cell atomistic MD simulations remain prohibitively expensive to run over cellular length (∼*μ*m) and time (∼seconds) scales. For example, a (100 nm)^3^ cubic cytoplasmic volume contains on the order of 10^8^ particles, so that, even with the most powerful computational resources available today, simulation timescales remain limited to the sub-millisecond time regimes.^145,146^ The accessible timescales can be extended by using enhanced sampling methods and Markov state modeling^147^ or by reducing the number of spatial degrees of freedom through coarse graining approaches.^148^ However, such models have to be parameterized/validated by comparison to experimental data and/or all-atom MD simulations that are themselves consistent with experimental results.

Recent all-atom simulations have shed light on how cellular environments may affect the diffusive properties of proteins in intracellular aqueous environments^31,145^ and near phospholipid bilayers.^146^ The results from these simulations have shown that, at very short time scales (less than 50 ns), proteins do not diffuse as isolated monomers, but instead form transient clusters with proteins that they encounter in their vicinity,^31^ an effect also seen in colloids, where cluster formation emerges due to the combined effects of short-range attraction and long-range repulsion.^149,150^ Transient cluster formation with constantly exchanging binding partners on sub-microsecond time scales,^31,145^ as well as volume exclusion effects due to molecular crowding^151^ and hydrodynamic interactions^152^ all contribute to an overall decrease in protein diffusivity as a function of increasing biomolecular volume fraction. All-atom molecular dynamics simulations of concentrated villin headpiece solutions showed that sizes of transient clusters increases on average with increasing biomolecular volume fraction. While different-sized clusters display different diffusion coefficients, the ensemble-averaged mean-square displacement increased linearly with time on sub-100 ns time scales at all simulated concentrations (consistent with Brownian diffusion). It stands to reason that Brownian diffusion would also manifest at microsecond and longer time scales, over which diffusive state transitions of a single villin headpiece protein engaging in transient cluster formation are averaged out. The authors also noted the transient cluster formation vastly diminishes the cage effect that produces distinguishable short and long-time diffusive regimes in hard sphere colloids. A more recent 10 *µ*s all-atom simulation of concentrated ternary mixtures containing villin, protein G, and ubiquitin showed that these proteins do not specifically interact with membranes, which creates a protein depletion zone in the immediate vicinity of the membrane for this particular simulation.^146^ Individual proteins that enter the depletion zone by chance can however diffuse faster near the membrane due to decreased molecular crowding and, consequently, decreased protein–protein contacts and clustering in that region. All-atom MD simulations have however not yet addressed how protein diffusion coefficients scale with molecular weight in the *μ*s – ms regime. In addition to computational resource limitations that prevent access to *μ*s – ms time scales, another critical challenge for molecular dynamics simulations is the selection and parameterization of force fields that balance the strengths of protein–protein and protein–water interactions. The strengths of these interactions strongly affect protein structures and protein aggregation, and thereby protein diffusion, and further developments are needed to match all of the available experimental data.^153^ Because of these challenges, a quantitative theoretical foundation that could explain the experimentally observed deviations from the Stokes–Einstein diffusion theory is not yet available.

To go beyond the current limitation of 10 *µ*s simulation times accessible by all-atom MD simulations, protein docking algorithms based on Markov Chain Monte Carlo (MCMC) sampling were used to efficiently determine protein spatial coordinates corresponding to low energy arrangements in crowded environments.^147^ The reduced computational cost of this approach enabled simulation times of several seconds while retaining all-atom detail within the simulated volume. Intriguingly, and in qualitative agreement with the experimental results discussed above, the simulation results showed that the rate of the diffusion coefficient slowdown (relative to the fastest diffusion rate) did not scale linearly with the size/radius of the protein (as predicted by the Stokes–Einstein diffusion theory). Instead, the rate of diffusion coefficient slowdown trended toward an exponential dependence, as the macromolecular crowding was increased toward a physiologically relevant volume fraction of 30%. This observation suggests that the amount of deviation from Stokes–Einstein scaling is dependent on the macromolecular density. The authors did not explore the molecular origins of this deviation further, attributing it instead to “possibly reflecting the complexity and heterogeneity of the dense protein solutions.” Running additional simulations with mixtures of different types of proteins could perhaps provide more specific insights. From a more practical perspective, the above findings strongly suggest that the experimentally observed variation in scaling exponents *β* (which range from 0.54 to 0.8) could be due to varying cytoplasmic densities among different bacterial species cultivated under different environmental conditions. Future experiments are needed to test this hypothesis quantitatively.

## VI. CONCLUSION

In summary, we reviewed currently available single-molecule tracking analysis algorithms designed to resolve diffusive states of proteins and protein complexes in living cells. We described several experimental approaches that enable the assignment of resolved diffuse states to protein complexes of defined composition. Moreover, currently available diffusion coefficient data for freely diffusing proteins and protein complexes of different molecular weights show that as molecular weight increases, protein diffusion rate slows more than what is predicted by the Stokes–Einstein diffusion theory, at least in bacterial cells. The scaling relationship between diffusion coefficient and molecular weight seems to be dependent on molecular density in the cytoplasm, but further experiments are necessary to quantify this dependence more completely. Such measurements will provide important benchmarks for computational simulations of conformational, translational, and rotational protein dynamics in intracellular aqueous environments.

## Data Availability

Data sharing is not applicable to this article as no new data were created or analyzed in this study.
